# Solid Phase Extraction Purification of Saliva Samples for Antipsychotic Drug Quantitation

**DOI:** 10.3390/molecules23112946

**Published:** 2018-11-12

**Authors:** Ewelina Dziurkowska, Marek Wesolowski

**Affiliations:** Department of Analytical Chemistry, Medical University of Gdansk, Gen. J. Hallera 107, 80-416 Gdansk, Poland; marwes@gumed.edu.pl

**Keywords:** saliva, solid phase extraction, neuroleptics, metabolites

## Abstract

Saliva is far less popular as a diagnostic material than blood. This has resulted in a lack of procedures for the sampling and handling of saliva, e.g., effective ways to purify endogenous compounds from saliva to enable a simultaneous determination of xenobiotics such as neuroleptics. Therefore, the aim of this study was to develop an analytical procedure to purify saliva samples so that it is then possible to simultaneously determine five neuroleptics (aripiprazole, clozapine, olanzapine, quetiapine and risperidone) and the antiepileptic drug carbamazepine, and their respective metabolites (dehydroaripiprazole, *N*-desmethylclozapine, *N*-demethylolanzapine, norquetiapine, 9-*OH*-risperidone and carbamazepine-10,11-epoxide). A study of three types of solid-phase extraction (SPE) columns showed that the purest eluates were obtained using columns containing ion exchange sorbent. The sorbents were first washed with water then with a mixture of water and methanol (1:1), and the adsorbed residue was eluted with a 5% ammonia solution in methanol. Saliva samples for SPE were diluted with 2% formic acid and a mixture of methanol and water (1:1). This procedure was developed to purify a saliva sample spiked with a mixture of neuroleptics and carbamazepine, and their respective metabolites. A chromatographic analysis confirmed the isolation of all compounds, indicating that this procedure can be used in further development and validation for a method designed to monitor the levels of neuroleptic drugs in saliva and to monitor their uptake by patients.

## 1. Introduction

Solid phase extraction (SPE) is one of the most frequently used methods of sample purification and isolation of selected substances. Although blood is one of the most frequently tested biological matrices, inconveniences associated with blood sampling such as the possibility of infection and the required presence of qualified medical staff, are reasons why other alternative biological matrices are being considered. One of the alternatives is saliva, being incomparably more accessible and easier to collect than blood. As saliva sampling is non-invasive, monitoring the concentration of analytes in saliva is recommended for children, the elderly and those who may not cooperate with the physician. In addition, the presence of qualified medical personnel is not required during saliva sampling.

As a test material saliva has several disadvantages. Sampling is not sterile, which means that the compounds to be determined may be degraded by microorganisms [1]; the concentration of the test substance(s) is usually lower than in blood and depends on many factors, including the molecular weight of the analyte, lipid solubility, pKa of the compound, the degree of binding to blood proteins or salivary flow; the concentration of a given substance is an individual feature of the tested person; saliva contains only the unbound forms of substances, i.e., active forms [2].

The use of saliva in the analysis of active compounds is not common. Therefore, there are no uniform procedures to determine how to sample saliva, store it and determine the substance being tested. In addition, the preparation of the sample requires appropriate treatment if it has to be analyzed chromatographically. Frequently, researchers test saliva with the same procedure as the determination of compounds in blood, but due to the specific nature of saliva, appropriate modifications are necessary [2].

Compounds determined in saliva are mainly steroid hormones, as well as non-steroid hormones, such as melanin or thyroid hormones. In addition, saliva is also used in screening for neoplastic lesions occurring in the oral cavity, the head and neck. A precondition for the use of saliva in quantitative analysis is the correlation between blood and saliva concentrations [3]. Such correlations have been found for antiepileptic drugs [4,5,6,7] and antipsychotics [8,9,10,11].

Saliva, as an easily accessible research material, can be a good source of information about the medications administered to mentally ill people. This applies especially to patients who do not cooperate with a doctor, as well as those who are overly stimulated as a result of discontinuation of drugs or independent modification of therapy. In addition, as a result of polypragmasy, i.e., combined therapy, interactions between the drugs used may occur, so a quick and suitably sensitive method of monitoring the concentration is needed. A simple and non-invasive method is to determine their concentration in saliva. In the case of discontinuation of drugs, it is also advisable to determine the level of their metabolites in addition to the parent substances.

The isolation of neuroleptics from saliva is usually performed with liquid-liquid extraction (LLE) [12,13,14,15,16]. However, the solvents used for extraction must differ in polarity so that the phases do not mix and can therefore be separated at a later stage. The use of solid phase extraction avoids the use of such solvents. Also the volume of solutions used for extraction is usually smaller.

Literature data only indicate a few cases of using SPE for the determination of neuroleptics in saliva [11,17]. The drugs extracted from saliva using SPE include older generation antiepileptic drugs like phenytoin [18] and valproic acid [19]. However, there is no information on the use of SPE for the isolation of carbamazepine. The literature also shows that there is no established method for simultaneous analysis of neuroleptics and carbamazepine in saliva from polypragmasy patients.

As already mentioned, due to the lower popularity of saliva as a diagnostic material, there are no standard procedures for its sampling, storage or preparation for isolation of compounds contained in saliva. The analysis of literature data does not make it clear which of the procedures may be useful for the determination of neuroleptics or antiepileptic drugs in saliva. In addition, there are also no publications on the purification of blank saliva samples to be able to compare the effects of the different types of solvents used in the individual extraction steps on the purification of diagnostic material.

Taking the above into account, this study developed a method for purifying endogenous substances from saliva so that it was possible to quickly and reliably determine the most commonly used neuroleptics (aripiprazole, clozapine, olanzapine, quetiapine and risperidone) and carbamazepine, along with their respective metabolites (dehydroaripiprazole, *N*-desmethyl-clozapine, *N*-demethylolanzapine, norquetiapine, 9-*OH*-risperidone and carbamazepine-10,11-epoxide) in this diagnostic material.

## 2. Results and Discussion

Although saliva is a readily available test material and in some cases can replace blood during monitoring of both endogenous compounds and xenobiotics, it requires a different preparation for analysis due to the specific proteins present in it, in particular glycoprotein [2]. Therefore, this study was undertaken to select the best procedure for saliva purification with SPE, with the intention of developing a method allowing the determination of five neuroleptics, carbamazepine and their respective metabolites.

### 2.1. Solid Phase Extraction

Solid phase extraction makes it possible to isolate compounds from a biological matrix by adsorption of the analyte on an appropriately selected sorbent. For this purpose, a liquid sample of the biological material is diluted with a solution with a predetermined pH to enhance the interaction between the analyte and the sorbent. In addition, a methanol or acetonitrile solution is usually added to the sample to precipitate the proteins. Both protein precipitation and saliva dilution reduce the viscosity of the saliva. The eluate is evaporated by flux with nitrogen, the dry residue is then dissolved in a mobile phase and chromatographed. The chromatographic separation was carried out on the extracts of blank saliva samples in which we observed the residues of the matrix eluted from the column in the elution process.

In order to achieve the objective of the study, we selected the composition of the solution used to dilute the saliva samples prepared for extraction, the type of the sorbent for the purification of samples, and the type of solvents and their mixtures for washing the sorbent after loading the sample. We also investigated the effect of pH on the elution of residues. Detailed data on the compositions of the solvents used and their mixtures used for sample dilution, sorbent washing and elution of analytes, are presented in Table 1.

### 2.2. Extraction Columns

Three types of columns were selected for the study: Strata-X (I), intended for simultaneous extraction of compounds with different polarities, Strata-X-C (II) and Strata-X-CW (III) containing a strong or weak cation exchange sorbent, respectively, for analysis of weak bases (all from Phenomenex, Torrance, CA, USA). The selection of these three types of columns was based on literature data indicating that columns used for saliva purification before the determination of risperidone and its metabolite contained strong cation exchangers as sorbents [11], and that columns with the C_18_ sorbent intended for lipophilic compounds analysis were used for the simultaneous determination of e.g., risperidone, clozapine, quetiapine and aripiprazole [17]. We also took into account the chemical properties of the neuroleptics to be isolated from the saliva using our method of sample purification. In addition, for preliminary selection of columns, a Phenomenex software (https://www.phenomenex.com/Tools/SPEMethodDevelopment; Torrance, CA, USA) was used.

### 2.3. Optimization of the SPE Procedure

Optimization of the SPE procedure was carried out in several stages. The first stage included determination of the influence of the solvent pH and its composition on the process of washing the sorbent after the loading of samples. A mixture of water and methanol (1:1) was used to dilute the samples. Methanol (procedures 1–4) or acetonitrile (procedures 5–8) were used to elute the residues of endogenous compounds from the column (Table 1). The next stage of optimization was to determine the effect of the pH of the elution solvent on the efficiency of saliva purification from the endogenous compounds. For this purpose, formic acid was added to both elution solvents methanol and acetonitrile so that the final concentration of the solution was 5% (procedures 9–16, Table 1). Continuing the study, ammonia solution was added to the eluent so that its final concentration was 5% (procedures 17–24, Table 1). In procedures 9–24, the composition of the mixture for sample dilution and the washing solvents remained the same as in procedures 1–4. Each of the 24 procedures was performed using the three types of column—Strata-X, Strata-X-C and Strata-X-CW.

#### 2.3.1. Sorbent Washing

**Figure 1:**
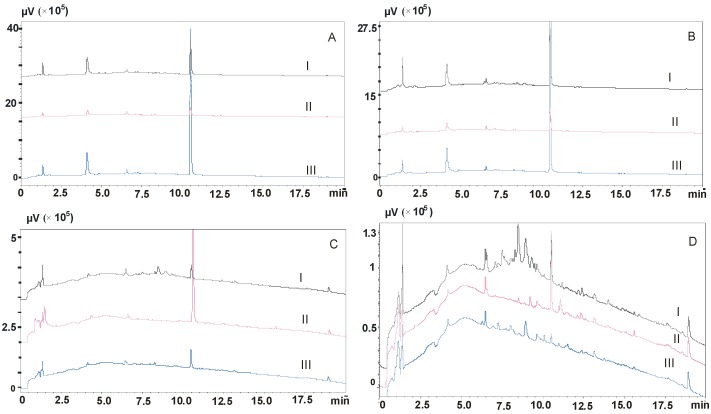
Chromatograms of blank saliva samples after elution of column with methanol. (**A**)—procedure 1, (**B**)—procedure 2, (**C**)—procedure 3, (**D**)—procedure 4; I—Strata X columns, II—Strata X-C columns, III—Strata X-CW columns.

In the first stage of the study, the effect of the pH of washing solvents was evaluated (procedures 1–8, Table 1). Washing with water or 2% formic acid did not ensure a satisfactory purification of the sample regardless of the type of columns used (procedures 1 and 2, Figure 1A,B). Washing with a mixture of water and methanol slightly reduced the degree of eluate contamination, so that the intensity of endogenous compound peaks decreased, but they were still visible on chromatograms (procedures 3 and 4, Figure 1C,D). It was not possible to purify the samples thoroughly.

**Figure 2:**
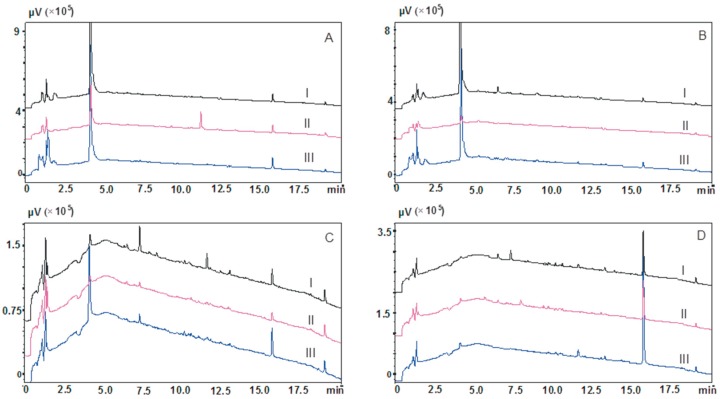
Chromatograms of blank saliva samples after elution of column with acetonitrile. (**A**)—procedure 5, (**B**)—procedure 6, (**C**)—procedure 7, (**D**)—procedure 8; I—Strata X columns, II—Strata X-C columns, III—Strata X-CW columns.

Similarly, in procedures 5 and 6, water or 2% formic acid did not purify the sample. Although the intensity of peaks reflecting the presence of endogenous compounds was lower, their presence in the same retention times as some less lipophilic compounds, e.g., olanzapine and its metabolite may mask the analytes’ peaks and not allow their detection (Figure 2B). The Strata-X-C columns were the exceptions to this, which significantly reduced the elution of impurities appearing in the initial period of chromatographic analysis (procedure 6, Figure 2(B-II)).

Washing with a mixture of water and methanol reduced or eliminated impurities that manifested as peaks in the initial section of the chromatogram (retention time approx. 4 min). The Strata X-CW column was the exception where in procedure 7 the peak peak with retention time about 4 min. on the chromatogram had a high intensity (Figure 2(C-III)).

The use of a mixture of water and methanol (procedure 7) as well as the two-stage procedure of elution (procedure 8) did not result in a satisfactory purification of extracts (Figure 2D). Despite the use of a mixture of solvents with greater eluting power for lipophilic impurities, peaks of endogenous compounds were still observed on the chromatogram, which may prevent the analysis of saliva of patients treated with e.g., carbamazepine or aripiprazole.

#### 2.3.2. Residue Elution

The next stage of the study was to optimize the pH of the eluent used in the last stage of the SPE. For this purpose, the pH of both the methanol and acetonitrile was modified with formic acid (procedures 9–16, Table 1) or ammonia (procedures 17–24, Table 1).

In the case of procedures 9–12 in which 5% formic acid solution in methanol was used for elution, the least effective procedures were those in which aqueous solutions were used for washing. Peaks of endogenous compounds with short retention times (Figure S1A,B) were still observed on the chromatograms. The best results of sample purification were obtained when a mixture of methanol and water was used to wash the columns (procedure 11, Figure S1).

In addition, two-stage washing (procedure 12, Figure S1) and the use of Strata-X-C and Strata-X-CW columns resulted in a satisfactory purification of extracts, as shown in Figure S1D-II,D-III. Similarly, in the case of the use of 5% formic acid in acetonitrile (procedures 13–16), the least effective methods of saliva purification were those where aqueous solutions were used to wash the sorbent, and in particular, when procedures 13 and 14 were performed with the use of Strata-X or Strata-X-CW columns. Endogenous peaks were observed on chromatograms throughout the analysis and had high intensity (Figure S2A,B). The most satisfactory results for saliva purification were achieved when washing the sorbent with methanol and water (procedure 15, Figure S2C) or two-stage washing (procedure 16, Figure S2D). Also in this case the ion exchange columns (Figure S2(C-III,D-II,D-III)) turned out to be the most suitable.

Comparing chromatograms for procedures in which 5% formic acid solution in methanol (procedures 11, 12) or acetonitrile (procedures 15, 16) were used for elution, the impression was that the use of acetonitrile resulted in better saliva purification. However, it should be borne in mind that most analytes are better soluble in methanol than acetonitrile. Assuming that the most efficient extraction procedure will be used to develop a method for the determination of neuroleptics in saliva, the use of acetonitrile for the elution of analytes from the sorbent may reduce the extraction efficiency, despite good purification of the sample from endogenous compounds. The accuracy of the developed method will then be lower.

The next stage of optimization of the extraction process was to examine the effect of alkaline pH of the eluent on eluate purification. Before sample extraction, samples were diluted only with a mixture of methanol and water (1:1). To wash the sorbents, four solutions were used (water, 2% formic acid solution, methanol/water mixture (1:1) as well as two-stage washing, while elution was performed with 5% ammonia solution in methanol or acetonitrile (procedures 17–24).

The use of aqueous solutions for washing the sorbent did not enable the purification of any of the examined types of columns. All chromatograms showed numerous high intensity peaks indicating the presence of endogenous compounds (Figure S3A,B). Optimization of the pH of methanol ensured satisfactory results of sample purification, but only when a methanol/water mixture was used to wash the Strata X-C columns (procedures 19 and 20).

Low intensity peaks associated with the presence of endogenous compounds occurred only in the initial section of the chromatogram (Figure S3(C-II,D-II)). The use of 5% ammonia solution in acetonitrile as an eluent (procedure 21–24) did not give satisfactory results even when a mixture of methanol and water had been used to wash the sorbent. In this case, the intensity of peaks from endogenous compounds whose retention times (in area 2.5–20 min) were similar to those of neuroleptics or carbamazepine decreased, but not to a sufficient degree (Figure S4).

#### 2.3.3. Dilution of Samples

Analysis of HPLC chromatograms showed the most effective procedure was washing the sorbent in two stages, by washing the columns with water followed by using a mixture of methanol and water (1:1). In addition, the fewest peaks associated with the presence of endogenous substances were observed for Strata-X-C and Strata-X-CW columns, and therefore they were used for further studies. The washing process was carried out in two stages, while methanol or acetonitrile solutions with a pH modified with formic acid or ammonia (procedures 25–28) to elute the residues.

**Figure 3:**
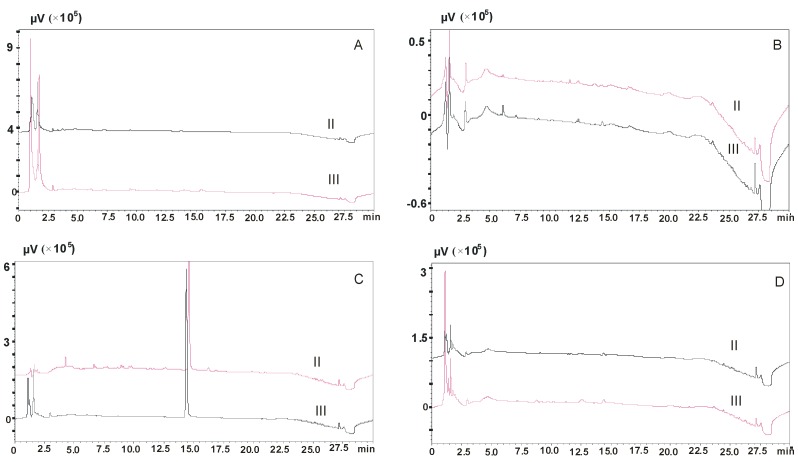
Chromatograms of blank saliva samples after elution of column with modification of the pH of the sample dilution solution and the elution solution. (**A**)—procedure 25, (**B**)—procedure 26, (**C**)—procedure 27, (**D**)—procedure 28; II—Strata X-C columns, III—Strata X-CW columns.

In addition, the effect of the pH of the solution used to dilute the sample was also determined by adding 1 mL of 2% formic acid solution (procedures 25, 27). A detailed description of the composition of solutions used in procedures 25–28 is presented in Table 1. Analysis of chromatograms showed that the most effective saliva purification procedure was procedure 25 in which, in addition to the mixture of methanol and water, 2% formic acid solution was used to dilute the samples. A 5% ammonia solution in methanol was used also to elute the adsorbed residues, as shown in Figure 3. In addition, a satisfactory purification effect was also obtained using procedure 28.

#### 2.3.4. Selection of the Optimal SPE Procedure

**Figure 4:**
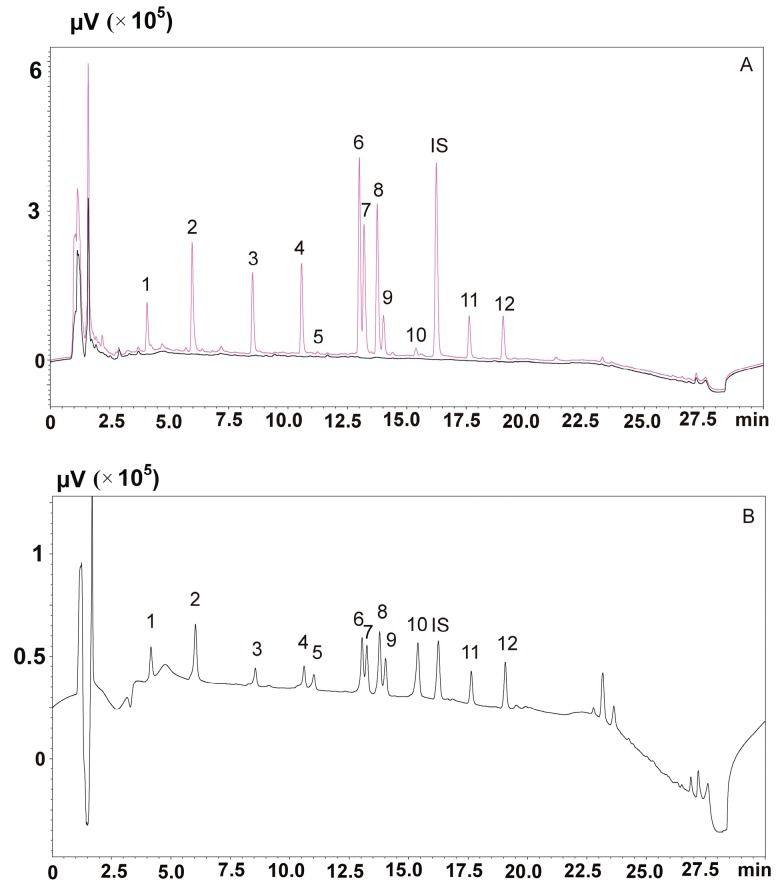
Chromatograms of a mixture of solutions of neuroleptics, carbamazepine and their metabolites. (**A**)—saliva extract spiked with a mixture of analytes (100 ng/mL), obtained with the use of Strata X-C columns and the procedure 25. (**B**)—mixtures of standard solutions (20 ng/mL): 1—*N*-demethylolanzapine, 2—olanzapine, 3—9-*OH*-risperidone, 4—risperidone, 5—carbamazepine-10,11-epoxide, 6—*N*-desmethylclozapine, 7—norquetiapine, 8—clozapine, 9—quetiapine, 10—carbamazepine, IS—chlordiazepoxide, 11—dehydroaripiprazole, and 12—aripiprazole.

The next stage of the study was to determine which of the four procedures (25–28) would enable the isolation from saliva of the most commonly used neuroleptics (aripiprazole, clozapine, olanzapine, quetiapine and risperidone) and antiepileptic drug carbamazepine, and their respective metabolites (dehydroaripiprazole, *N*-desmethylclozapine, *N*-demethylolanzapine, norquetiapine, 9-*OH*-risperidone and carbamazepine-10,11-epoxide). For this purpose, saliva was spiked with a mixture of standards so that their final concentrations were 100 ng/mL and then purified using procedures 25–28 and the Strata-X-C and Strata-X-CW columns. Procedure 25 and the Strata-X-C columns proved to be the most effective, enabling the isolation of all compounds, as presented in Figure 4. The analysis was carried out at 240 nm.

Our results clearly confirmed that the most effective purification of saliva samples was achieved by means of columns containing an ion exchanger. This type of sorbent also allowed all analytes to be isolated from the spiked saliva sample. The recovery results for analytes obtained using procedure 25 are presented in Table 2.

## 3. Materials and Methods

### 3.1. Chemicals and Solvents

Methanol, acetonitrile, formic acid and ammonia were manufactured by POCh (Gliwice, Poland). Triethylamine (TEA) was obtained from Sigma-Aldrich (St. Louis, MO, USA). All reagents were characterized by HPLC super grade purity. Deionized water was purified by Ultra-Toc/UV, Hydrolab (Straszyn, Poland).

### 3.2. UHPLC-DAD Analysis

Chromatographic separation was carried out using a Nexera XR UHPLC liquid chromatograph (Shimadzu, Kyoto, Japan), equipped with a CBM-20Alite control system, CTO-20AC thermostat, LC-30AD pump, SIL-30AC autosampler, SPD-M30A UV-VIS detector with diode array and highly sensitive measuring cell SPD-M30A (85 mm). A Luna Omega 3 µm column (Phenomenex, LC Column 150 × 3.0 mm ID) with Polar C_18_ 100 packing and a pre-column (Polar C_18_, 4 × 2.0 mm ID) were used. Saliva extracts were eluted at 35 °C with a mobile phase flow of 0.6 mL/min. The mobile phase was a binary system consisting of water with formic acid with 0.1% TEA (solvent A, pH 4.2) and acetonitrile (solvent B) with a gradient program starting from 85:15 (*v*/*v*) solvent A to solvent B, to 35:65 (*v*/*v*) solvent A to solvent B. The gradient program is presented in Table 3.

### 3.3. Collection and Pretreatment of Oral Fluid Samples

Saliva was collected using Salivettes^®^ (Sarstedt, Nümbrecht, Germany) or by direct placement in a plastic tube. Volunteers were required to not eat for at least half an hour prior to sampling and to rinse their mouths with water 10 min before taking the sample. Salivettes^®^, after being placed in the oral cavity, were chewed for 2 min, then centrifuged at 8000 rpm for 5 min. The obtained samples were frozen and stored at −20 °C until analysis.

### 3.4. Solid Phase Extraction

The thawed saliva was centrifuged, then 1 mL of liquid was collected and transferred to test-tubes made of polypropylene. Appropriate solutions were then added in order to dilute the sample, and then mixed before being shaken for 20 min and centrifuged at 8000 rpm. Supernatant was loaded onto activated columns (2 mL of methanol and 2 mL of water). The sorbent was washed and dried for 10 min and residues were eluted according to the procedures described in Table 1. Eluate was dried at 37 °C by flux of nitrogen, after which the dry residue was dissolved in 100 μL of the mobile phase 90:10 (*v*/*v*) solvent A to solvent B and subjected to UHPLC analysis.

## 4. Conclusions

The study successfully developed an analytical procedure to purify saliva samples so that five neuroleptics (aripiprazole, clozapine, olanzapine, quetiapine and risperidone) and carbamazepine, and their respective metabolites (dehydroaripiprazole, *N*-desmethylclozapine, *N*-demethyl olanzapine, norquetiapine, 9-*OH*-risperidone and carbamazepine-10,11-epoxide) could be simultaneously determined. This research used three types of SPE columns and showed that the most effective were those containing ion exchange sorbents.

Moreover, the most effective procedure was the one in which the saliva samples were diluted with 2% formic acid solution and methanol/water mixture (1:1). Water, and a mixture of water and methanol (1:1), were used to wash the column, while residues adsorbed on the columns were eluted with 5% ammonia solution in methanol. The purification procedure developed for saliva samples was used to purify saliva spiked with a mixture of neuroleptics, carbamazepine and their respective metabolites. This procedure confirmed the isolation of all the examined compounds, so can be used for further development and validation of a method designed to monitor the level of neuroleptics and monitor their uptake by patients in an easily accessible biological material, i.e., saliva.

## Figures and Tables

**Table 1:** Procedures used to examine the efficacy of solid phase extraction of saliva.

Procedure	Dilution Solvent (1 mL)	Washing Solvent (2 mL)	Elution Solvent (2 mL)
1	Methanol:Water (1:1)	Water	Methanol
2		2% formic acid	
3		Methanol:Water (1:1)	
4		Water; Methanol:Water (1:1)	
5	Methanol:Water (1:1)	Water	Acetonitrile
6		2% formic acid	
7		Methanol:Water (1:1)	
8		Water; Methanol:Water (1:1)	
9	Methanol:Water (1:1)	Water	5% formic acid in methanol
10		2% formic acid	
11		Methanol:Water (1:1)	
12		Water; Methanol:Water (1:1)	
13	Methanol:Water (1:1)	Water	5% formic acid in acetonitrile
14		2% formic acid	
15		Methanol:Water (1:1)	
16		Water; Methanol:Water (1:1)	
17	Methanol:Water (1:1)	Water	5% ammonium in methanol
18		2% formic acid	
19		Methanol:Water (1:1)	
20		Water; Methanol:Water (1:1)	
21	Methanol:Water (1:1)	Water	5% ammonium in acetonitrile
22		2% formic acid	
23		Methanol:Water (1:1)	
24		Water; Methanol:Water (1:1)	
25	Methanol:Water (1:1) 2% formic acid	Water; Methanol: water (1:1)	5% ammonium in methanol
26	Methanol:Water (1:1)		5% formic acid in methanol
27	Methanol: water (1:1) 2% formic acid		5% ammonium in acetonitrile
28	Methanol:Water (1:1)		5% formic acid in acetonitrile

**Table 2:** Recovery values for analytes under study.

Analyte	Recovery (%)	Analyte	Recovery (%)	Analyte	Recovery (%)
Aripiprazole	87.10	Olanzapine	89.30	Risperidone	91.13
Dehydro-aripiprazole	82.50	*N*-Demethyl-olanzapine	93.19	9-*OH*-Risperi-done	103.23
Clozapine	89.63	Quetiapine	92.51	Carbamazepine	68.77
*N*-Desmethyl-clozapine	89.46	Norquetiapine	85.60	Carbamazepine-10,11-epoxide	73.95

**Table 3:** UHPLC gradient program.

Time (min)	Gradient (Percentage of Solvent B by Volume)
0.01	17
0.5	17
17.0	40
20.0	65
25.0	90
25.2	17
30.0	17

## Supplementary Materials

Supplementary Materials are available online.
