# Supplementary material for: Solid Phase Extraction Purification of Saliva Samples for Antipsychotic Drug Quantitation
Source: Molecules. 2018 Nov 12;23(11):2946. doi: 10.3390/molecules23112946 (PMC6278404; doi:10.3390/molecules23112946)
Supplement: Supplementary file 1 [file molecules-23-02946-s001.pdf]

Supplementary Material

Solid Phase Extraction Purification of Saliva Samples for Antipsychotic Drug Quantitation

Ewelina Dziurkowska * and Marek Wesolowski

Department of Analytical Chemistry, Medical University of Gdansk, Gen. J. Hallera 107, 80-416 Gdansk, Poland; marwes@gumed.edu.pl
* Correspondence: elewajs@gumed.edu.pl; Tel.: +48-58-349-1097

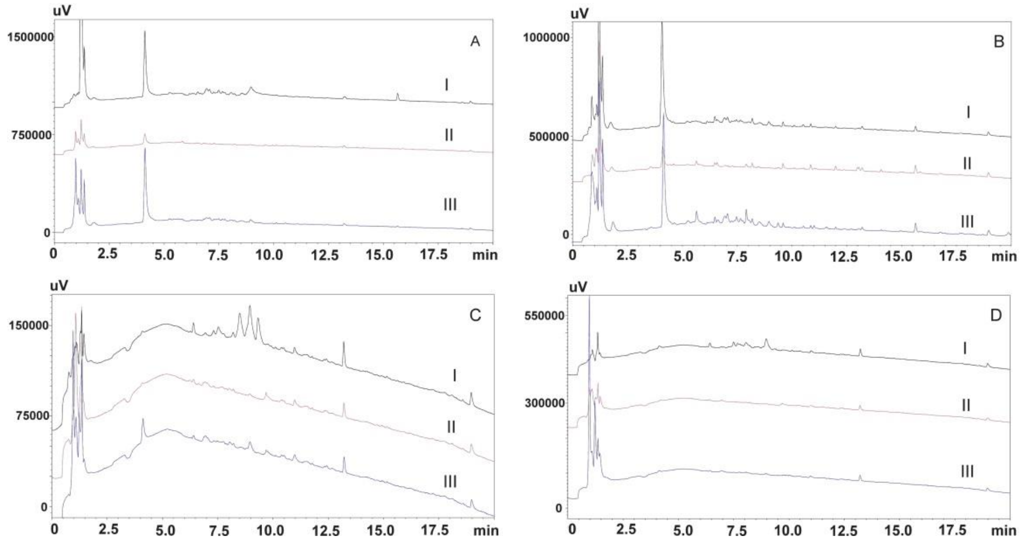

Figure S1. Chromatograms of blank saliva samples after elution of column with a 5% formic acid solution in methanol. (**A**)—procedure 9, (**B**)—procedure 10, (**C**)—procedure 11, (**D**)—procedure 12; I—Strata X columns, II—Strata X-C columns, III—Strata X-CW columns.

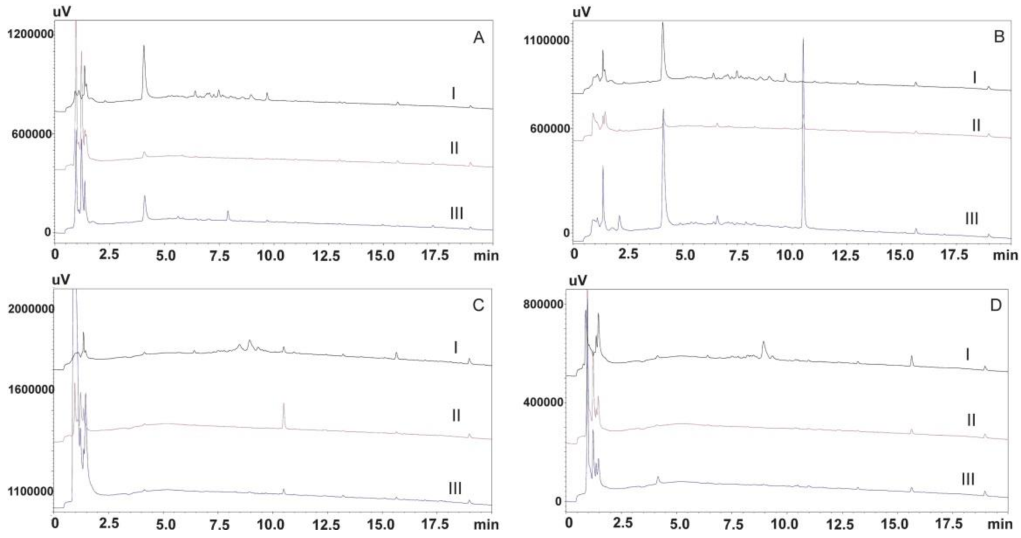

Figure S2. Chromatograms of blank saliva samples after elution of column with a 5% solution of formic acid in acetonitrile. (**A**)—procedure 13, (**B**)—procedure 14, (**C**)—procedure 15, (**D**)—procedure 16; I—Strata X columns, II—Strata X-C columns, III—Strata X-CW columns.

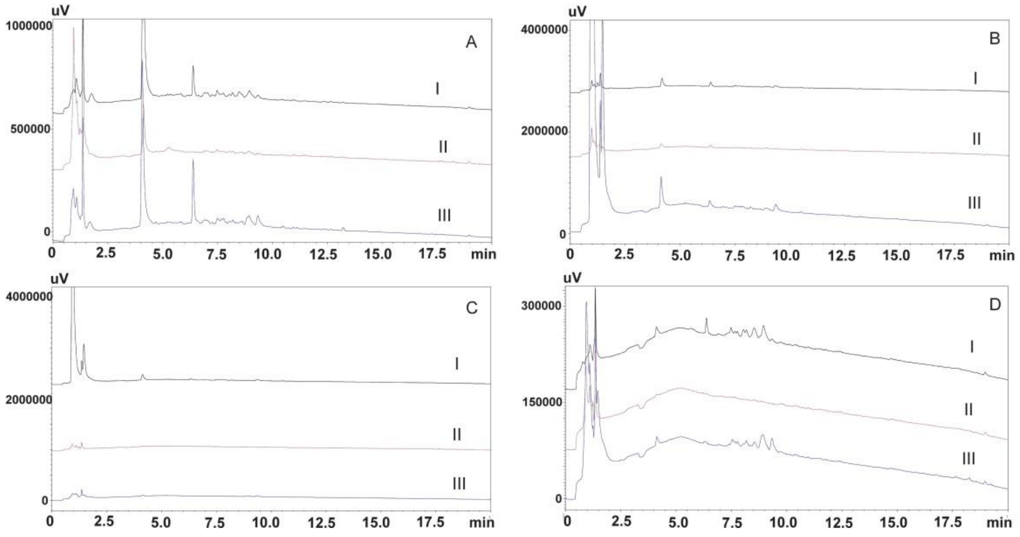

Figure S3. Chromatograms of blank saliva samples after elution of column with a 5% solution of ammonia in methanol. (**A**)—procedure 17, (**B**)—procedure 18, (**C**)—procedure 19, (**D**)—procedure 20; I—Strata X columns, II—Strata X-C columns, III—Strata X-CW columns.

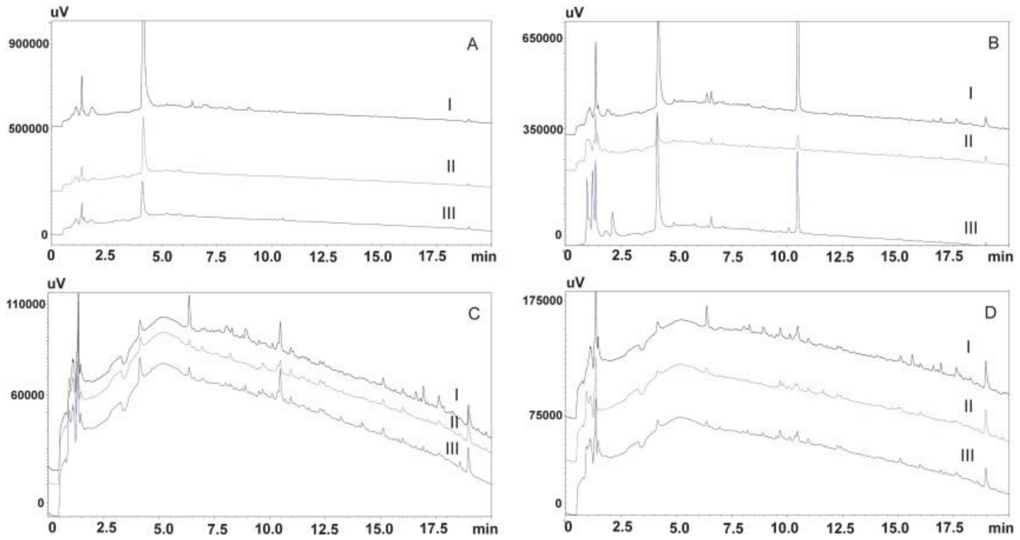

Figure S4. Chromatograms of blank saliva samples after elution of column with a 5% solution of ammonia in acetonitrile. (**A**)—procedure 21, (**B**)—procedure 22, (**C**)—procedure 23, (**D**)—procedure 24; I—Strata X columns, II—Strata X-C columns, III—Strata X-CW columns.
